# Intra-arterial Chemoperfusion for Lung Cancer Brain Metastases

**DOI:** 10.7759/cureus.108955

**Published:** 2026-05-16

**Authors:** Thomas J Vogl, Leon V Stein, Hamzah Adwan

**Affiliations:** 1 Clinic for Radiology and Nuclear Medicine, University Hospital Frankfurt, Goethe University, Frankfurt am Main, DEU

**Keywords:** brain metastases, intra-arterial chemoperfusion, lung cancer, response, survival

## Abstract

Purpose

The aim of this retrospective single-center study was to evaluate the safety and efficacy of palliative intra-arterial chemoperfusion (IAC) for patients diagnosed with recurrent or post-therapeutic progressive symptomatic lung cancer brain metastases (LCBM).

Materials and methods

A total of 10 patients (five women and five men; median age: 56 years (range: 40-80 years)) with recurrent or post-therapeutic LCBM were included. Chemotherapy consisted of either carmustine or a combination of mitomycin C, gemcitabine, and cisplatin. The radiological follow-up was performed using magnetic resonance imaging (MRI) according to the Response Evaluation Criteria in Solid Tumors (RECIST) 1.1. The overall survival (OS) was calculated using the Kaplan-Meier method.

Results

All patients were treated with prior systemic therapy, and seven patients additionally received radiation therapy. A total of 37 sessions of IAC (3.7/patient) were performed. No major complications occurred. The mean sum of the longest diameter (SLD) was 4.5±1.2 cm for all patients prior to treatment. The mean SLD for patients with radiological follow-up was 3.9±2.1 cm after treatment. Four patients (4/8) developed progressive disease. Three patients (3/8) achieved stable disease, and one patient (1/8) showed complete response. None of the patients had partial response. Two patients (2/10) were not assessable due to loss of radiological imaging post-interventional. The mean OS time was 9.7 months (95% CI: 2.4-17.0).

Conclusion

Although IAC seems to be a safe approach for palliative patients with recurrent or post-therapeutic progressive symptomatic LCBM, further studies with a larger patient population are needed to confirm its efficacy.

## Introduction

Lung cancer is mainly categorized into non-small cell lung cancer (NSCLC) and small cell lung cancer (SCLC) [1]. The 2021 World Health Organization (WHO) Classification of Lung Tumors emphasizes moving away from this division in favor of more precise subtyping especially for NSCLC [2]. Current diagnostic standards prioritize classifications integrated with molecular profiling [2]; nevertheless, the terms NSCLC and SCLC still appear in recent literature [3]. SCLC patients develop brain metastases more frequently compared to patients with NSCLC [4]. Among all lung cancer patients, 14.1% develop central nervous system metastases [5]. The prognosis of lung cancer brain metastases (LCBM) is poor, but it also depends on the subtype. Generally, the overall survival (OS) for LCBM patients is five months [5]. In studies that exclusively investigated NSCLC, the median OS was better at 12 months [6]. Recently, immunotherapy with tarlatamab demonstrated a significant extension of OS for patients with extensive-stage SCLC who had already received systemic therapy [7].

Treating LCBM involves both local and systemic strategies [3]. For NSCLC patients who are feasible, the approach is surgical resection of accessible metastases, followed by stereotactic radiosurgery (SRS) [3]. If surgery is not an option, therapy is dependent on driver mutations [3]. By comparison, whole brain radiotherapy is often used for patients with SCLC, possibly together with systemic platinum-based chemotherapy [3]. In NSCLC, systemic therapy for brain metastases has expanded to include immune checkpoint inhibitors and brain-penetrant targeted agents [3], while in SCLC, systemic treatment still relies mainly on platinum-based chemotherapy, with immunotherapy options recently approved for usage [3].

In this study, patients with LCBM were treated using a minimally invasive procedure called intra-arterial chemoperfusion (IAC). Administration of chemotherapeutic agents in IAC occurs through the supplying vessels of the brain [8]. The goal of IAC is to achieve higher concentration of drugs at the tumor site compared to systemic therapy [9,10]. Systemic exposure and its associated adverse effects may be reduced through the localized nature of this treatment approach [8].

Only few studies have explored the application of IAC in metastatic brain disease [11,12]. This study aims to add further evidence to the literature on IAC for patients with recurrent or post-therapeutic progressive symptomatic LCBM and to explore the safety and therapeutic efficacy of this treatment method.

## Materials and methods

Ethics

This retrospective study adhered to the ethics guidelines of the Declaration of Helsinki. Given the study's retrospective design, obtaining informed consent for participation was waived.

Inclusion and exclusion criteria

We retrospectively collected data from patients with recurrent or post-therapeutic progressive symptomatic LCBM who had been treated at the Clinic for Radiology and Nuclear Medicine of the Goethe University Hospital in Frankfurt between March 2010 and March 2024. Patients were selected based on individual clinical evaluation. The inclusion criteria were patients who had exhausted primary treatment options and subsequently received IAC with palliative intent. We excluded patients under 18 years of age and those with incomplete follow-up, specifically if tumor response evaluation according to the Response Evaluation Criteria in Solid Tumors (RECIST) could not be determined in addition to unknown date of death or last contact after the initial IAC treatment.

Treatment and follow-up

A comparable treatment approach had previously been described in our publications on primary malignancies [13] and brain metastases from breast cancer [14]. Intra-arterial chemotherapy consisted of either carmustine 50 mg/m² or a combination of mitomycin C 10 mg/m², gemcitabine 500-1300 mg/m², and cisplatin 50 mg/m². The IAC sessions were performed with an interval of four weeks. The chemotherapeutic drugs were administered after reaching the cervical segment of the internal carotid artery using various catheters including the sidewinder catheter, the cobra catheter, and the headhunter catheter. After the intervention, patients were required to maintain bed rest for several hours. A post-procedural computed tomography (CT) scan was performed to exclude complications, and patients were discharged in the absence of complications. Complications were categorized according to the Society of Interventional Radiology clinical practice guidelines [15]. A follow-up was carried out using magnetic resonance imaging (MRI). An MRI examination was performed before the start of the first IAC. Subsequently, another MRI was carried out every four weeks directly before the next IAC session. The sum of the longest diameter (SLD) of the target lesions was determined. Tumor response was classified according to the RECIST 1.1 [16].

Statistics

Statistical analyses were performed using IBM SPSS Statistics for Mac, Version 30 (IBM Corp., Armonk, New York, United States). The OS time was calculated using the Kaplan-Meier method from the date of the first IAC session until the date of death or the last follow-up. The OS of the subgroups was compared using the log-rank test. Categorical variables were presented as frequencies and continuous variables as mean and standard deviation or median and range.

## Results

Characteristics of patients and treatments

A total of 10 patients with LCBM were enrolled in our study, including five (5/10; 50%) women and five (5/10; 50%) men. The median age of the patients was 56 years (range: 40-80 years). Nine patients (9/10; 90%) had post-therapeutic progressive disease, and one patient (1/10; 10%) had recurrent disease. Initially, the number of LCBM was distributed among the three categories as follows: three patients (3/10; 30%) had 1-3 metastases, three (3/10; 30%) had 4-6 metastases, and four (4/10; 40%) had >6 metastases. A total of six patients, at a rate of 60%, were treated by two sessions of IAC or less. The remaining four patients were treated by three sessions of IAC or more at a rate of 40%. All patients had received systemic therapy, seven had received radiation, and none had undergone resection in the past. Five patients (5/10; 50%) were treated with carmustine, and five (5/10; 50%) were treated with a combination of mitomycin C, gemcitabine, and cisplatin. On average, 3.7 IAC sessions were performed per patient, resulting in a total of 37 IACs. The characteristics of the patient cohort are shown in Table 1.

**Table 1:** Characteristics of patients and treatments IAC: intra-arterial chemoperfusion

Parameter	Value
Number of patients	10
Median age (range)	56 years (40-80 years)
Sex	
Men n (%)	5 (50)
Women n (%)	5 (50)
Patients with 1-3 metastases n (%)	3 (30)
Patients with 4-6 metastases n (%)	3 (30)
Patients with >6 metastases n (%)	4 (40)
Initial situation	
Recurrent disease n (%)	1 (10)
Post-therapeutic progressive disease n (%)	9 (90)
IAC interventions	
Total	37
Per patient	3.7
Patients treated by 2 IAC sessions or less n (%)	6 (60)
Patients treated by 3 IAC sessions or more n (%)	4 (40)
Therapy prior to IAC	
Systemic therapy	10
Radiation	7
Resection	0

Complications

No major complications requiring treatment were observed among the patient cohort. Few patients complained about post-interventional headache at a rate of 30% (3/10), which was treated symptomatically.

Tumor response and survival

We were able to evaluate tumor response according to RECIST 1.1 in eight patients. Two patients could not be evaluated regarding tumor response due to loss of radiological imaging. The mean SLD for all patients was 4.5±1.2 cm before treatment, while the mean SLD for patients with radiological follow-up was 3.9±2.1 cm after treatment. A total of three patients (3/8) at a rate of 37.5% showed stable disease, and one patient (1/8) showed complete response at a rate of 12.5%. None of the patients had partial response. A total of four patients (4/8) developed progressive disease at a rate of 50%. Progressive disease was due to the occurrence of new metastases in three of four patients and in one patient because of the progression of the target lesions. The mean OS time was 9.7 months (95% CI: 2.4-17.0). The mean OS time for patients with four metastases or less was 11.7 months (95% CI: 0.12-23.4) compared to 4.3 months (95% CI: 2.1-6.6) in patients with five metastases or more (p=0.783). The main results regarding response and survival are summarized in Table 2.

**Table 2:** Oncological response and overall survival

Parameter	Value
Complete response n (%)	1 (12.5)
Stable disease n (%)	3 (37.5)
Progressive disease n (%)	4 (50)
Mean overall survival (months) for all patients	9.7 (95% CI: 2.4-17.0)

## Discussion

The poor prognosis of patients with LCBM [5] underlines the need for various and new therapeutic approaches. In our study, we evaluated the safety and efficacy of IAC for patients with recurrent or post-therapeutic progressive LCBM. Our patients were treated by a total of 37 IAC sessions leading to an encouraging local tumor control without major complications. The patients also achieved acceptable survival time. Additionally, we showed that patients with four metastases or less had a non-significant longer OS time compared to patients with five metastases or more.

A comparable study to ours is by Rong et al. [11], which included 54 patients with LCBM treated with IAC. Although their methodological approach is broadly similar, patients additionally received blood-brain barrier disruption, which may have influenced treatment response [11]. According to RECIST, Rong et al. reported zero complete responses, 30 partial responses, 16 stable diseases, and eight progressive diseases in patients with LCBM [11]. In contrast, patients in our study achieved one complete response, no partial response, three stable diseases, and four progressive diseases. Our patients had more than three metastases in 70% (7/10) of the cases compared to 62.9% (34/54) in the cohort of Rong et al. [11]. Furthermore, 50 mg teniposide, 25 mg nimustine, and 100 mg carboplatin were used differing from our study [11].

Another older study by Fortin et al. used IAC to treat LCBM in 12 patients [12]. In their study, blood-brain barrier disruption was applied in some patients, and different therapeutic agents were used [12]. Fortin et al. also reported better LCBM response rates according to the RECIST criteria compared to the present study [12]. The chemotherapy they applied was carboplatin. Differences in therapeutic agents or the application of blood-brain barrier disruption may explain the variation in radiological responses between the present and the two compared studies. These differences suggest that the lower response rates in our patients may be related to variations in treatment protocols and patient selection, highlighting the need for more consistent approaches in future studies.

Former high rates of adverse events have raised concerns regarding the safety of IAC [17]. More recent work, including our study, did not observe serious complications and supports the idea that IAC can be performed safely [18].

In the current study, progressive disease was primarily driven by the development of new metastases. This finding suggests that IAC may be effective against preexisting metastases but insufficient to prevent the emergence of new lesions [14]. To address this limitation, combining IAC with other treatment modalities, including radiation or systemic therapies, may be necessary [14]. As previously described, we sought to maximize regional coverage by administering the drugs into the C1 segment of the internal carotid artery rather than using a more selective approach [13,14].

The development of new therapeutic agents could significantly improve the efficacy of IAC, as proposed by the meta-analysis from Pinkiewicz et al. [19]. A new approach is to combine IAC with radioligands to target the molecular structures of tumor cells [20]. Although there are currently no large clinical studies or evidence regarding the efficacy of this approach [20], it demonstrates the potential and developmental possibilities of intra-arterial drug delivery in the treatment of brain malignancies.

This study has several limitations. Firstly, it is a retrospective single-arm study without a control group. Secondly, the number of patients included was too low to draw sufficient clear conclusions, especially regarding survival. Thirdly, we unfortunately did not have information regarding the histological subtypes of the lung cancer. Furthermore, Cox regression analyses could not be conducted due to the low number of events in the survival analysis. Additionally, there are missing data regarding previous therapy, specifically concerning the type of radiotherapy and the time between the last treatment and the start of IAC. Lastly, no data was available regarding the symptoms as well as the quality of life of the patients.

## Conclusions

Our study showed that patients with recurrent or post-therapeutic LCBM treated by IAC as a palliative treatment had no major complications, which indicates that IAC appears to be a safe therapy option. Additionally, our results showed that our patients achieved an encouraging local tumor control and acceptable survival, considering their high-tumor burden. However, the findings are mainly limited by the retrospective study design, the low number of patients, and missing data. This highlights the importance of further studies with larger and more detailed patient cohorts to confirm the safety and therapeutic efficacy of IAC for LCBM.
